# Paid Parental Leave Policies Among *U.S. News & World Report* 2020-2021 Best Hospitals and Best Hospitals for Cancer

**DOI:** 10.1001/jamanetworkopen.2021.8518

**Published:** 2021-05-11

**Authors:** Diana J. Lu, Benjamin King, Howard M. Sandler, Nancy J. Tarbell, Mitchell Kamrava, Katelyn M. Atkins

**Affiliations:** 1 Department of Radiation Oncology, Cedars-Sinai Medical Center, Los Angeles, California; 2 Department of Radiation Oncology, Massachusetts General Hospital and Harvard Medical School, Boston

## Abstract

This cross-sectional study examines paid parental leave policies for faculty and staff physicians at leading US hospitals and cancer centers.

## Introduction

The American Academy of Pediatrics endorses 12 weeks of paid parental leave on the basis of studies showing health benefits to parents and children.^1^ Parental leave policies can impact the distribution of child-rearing responsibilities, which may be associated with gender equity and retention of women in medicine.^2^ Although prior studies have examined leave policies at select top-tier US medical schools^3^ and graduate medical education–sponsoring institutions,^4^ there is a paucity of data describing paid parental leave for faculty and staff physicians at leading US hospitals and cancer centers, which was studied here.

## Methods

This cross-sectional study qualified as nonregulated, non–human participant research exempt from approval and the need for informed consent according to the Cedars-Sinai Medical Center institutional review board. This study follows the Strengthening the Reporting of Observational Studies in Epidemiology (STROBE) reporting guideline.

In October 2020, we reviewed publicly accessible websites to retrieve child-bearing and parental leave policies for the 2020 to 2021 *U.S. News & World Report* Top 20 Best Hospitals and Best Hospitals for Cancer. A total of 27 unique institutions were included in our analysis. Human resources and/or benefits representatives were contacted at each institution to verify policies and/or retrieve unavailable or incomplete policies. Duration of paid childbearing (maternity) and parental leave and extent of salary support was obtained. Parental leave included leave available after childbirth or disability (for birthing mothers) and for nonbirthing mothers, partners, and adoptive parents, as applicable. Paid leave was defined as receiving 50% or more of base salary pay and excluded regular accrued paid time off (eg, vacation); duration of paid leave less than 50% of salary or without pay was considered to be 0. For additional details, see eMethods in the Supplement. Mean durations of leave were calculated using Stata statistical software version 16.1 (StataCorp).

## Results

Of 27 unique institutions, 2 (7.4%) would not provide pay details during short-term disability and 1 (3.7%) would not confirm online information for accuracy. Six institutions (22%) offer longer leave and/or greater pay according to the extent of caregiving responsibility (eg, primary vs secondary; 2 institutions) or employment position (eg, academic faculty vs nonfaculty staff physician; 4 institutions) (Table).

**Table.** Paid Parental Leave at *U.S. News & World Report* 2020-2021 Best Hospitals and Best Hospitals for Cancer

Overall rank and cancer rank^a^	Duration of paid leave, wk		
	Total paid leave for birth mothers, childbirth plus parental leave at ≥50% base pay^b^	Childbirth leave for birth mothers^b^	Paid parental leave for nonbirth mothers, partners, and adoptive parents
Top 5			
Top 5	Could not be determined^c^	6 STD (would not disclose pay details)^d^	None
Top 5	12	8 Maternity (100% pay)	4 (100% pay)
Top 5	10^d^^,^^e^	6 STD (100% pay)^d^^,^^e^	4 (100% pay)^e^
Top 16-20	6	6 STD (0% paid; requires use of sick, VHT, PTO, or paid parental leave)^d^	Caregiver: primary, 6 (100% pay); secondary, 2 (100% pay)
Top 11-15	6^f^	6 STD (60% pay; maximum benefit $15 000/mo; 14-d waiting period)^d^^,^^f^	None
Top 6-10			
Top 16-20	8^g^	8 (100% pay)^g^	8 (100% pay)^g^
Top 6-10	6^h^	6 STD (100% pay; 7-d waiting period)^d^	None^h^
Top 6-10	6 (Nonfaculty); 12 (faculty)^f^	Nonfaculty: 6 STD (60% pay; maximum benefit $15 000/mo; 14-d waiting period)^d^^,^^f^	Nonfaculty: none
		Faculty: 12 (100% pay)	Faculty: 12 (100% pay; if have ≥50 caregiving responsibility)
Top 16-20	6	0 (Not eligible for STD)	6 (100% pay; requires exhaustion of sick leave)
Top 6-10	10^e^	6 STD (75% pay; 7-d waiting period; requires ≥6-mo employment duration)^d^	4 (100% pay)^e^
Top 11-15			
>20	12	6 (100% pay; if >6 wk due to medical necessity, pay is via extended sick time, short-term sick time, or PTO)	6 (100% pay)
Top 6-10	8^g^	8 (100% pay)^g^	8 (100% pay)^g^
>20	14 (Faculty)^e^; 8 (nonfaculty)	6 STD^d^ (faculty, 100% pay^e^; nonfaculty, voluntary disability insurance^d^ 60% pay; maximum weekly benefit $1300)	8 (6 wk at 100% pay; 2 wk at 60% pay, supplemented with sick, VHT, or PTO)
>20	6^i^	6 STD^d^ (100% pay, no waiting period)	None^i^
>20	10 (Faculty)^e^; 0 (nonfaculty)	6 STD (faculty, 100% pay; nonfaculty, supplemental policy, 60% pay; maximum weekly benefit $1000; sick time must be exhausted)^d^	4 (Faculty, 100% pay)^e^; None (nonfaculty)
Top 16-20			
>20	Could not be determined^c^	6 STD (would not provide pay details)^d^	None
>20	0	6 STD (75% pay; maximum weekly benefit $2000; 14-d waiting period)^d^	None
Top 11-15	12	12 (100% pay) for primary caregiver (per department chair discretion)	Up to 12 (100% pay) for the primary caregiver (per department chair discretion)
>20	6^h^	6 STD (supplemental policy; 1 wk at 100% pay per year of employment up to 10 wk, then 80% of base pay)^d^^,^^j^	None^h^
Top 16-20	8	6 STD (50%-66.7% pay depending on length of employment; requires 7-d waiting period)^d^	2 (100% pay; requires ≥6 mo employment duration)
NA			
Top 5	6	6 (50% pay; 7-d waiting period; must exhaust sick time)	None
Top 5	12	6 STD (100% pay; no waiting period)^d^	6 (100% pay)
Top 6-10	0^k^	6 (0% pay; can use accrued PTO, sick, VHT, and/or discretionary leave up to 6 d/y)	None^k^
Top 11-15	10	6 STD (50%-66.7% pay, depending on plan; no waiting period)^d^	4 (100% pay)
Top 11-15	0^d^^,^^c^^,^^i^	STD (50% pay; weekly maximum $961.54; 14-d waiting period)^d^^,^^c^^,^^l^	None^i^
Top 11-15	6^h^	6 STD (60% pay; maximum weekly benefit $3462; 7-d waiting period)^d^	None^h^
Top 11-15	10 (Faculty); 6 (nonfaculty)	6 STD (100% pay)^d^	Faculty: 4 (100% pay); nonfaculty: 0

Abbreviations: NA, not applicable to rank or not ranked; PTO, paid time off; STD, short-term disability; VHT, vacation and holiday time.

^a^ Hospital rankings are designated by groups of 5 or if more than 20. For full list of included institutions, see eMethods in the Supplement.

^b^ Uncomplicated vaginal delivery time was used for leave estimates.

^c^ Human resources or benefits department confirmed parental leave policy and availability of STD but would not confirm STD pay details.

^d^ STD length is typically 6 weeks for vaginal delivery and 8 weeks for cesarean delivery, with possible extension beyond 8 weeks for medical necessity.

^e^ Requires at least 1-year employment duration and at least 1250 hours at institution.

^f^ Requires enrollment in voluntary STD plan at time of initial eligibility; institution does not participate in state disability insurance. Institution has approved a new paid family leave benefit for faculty and staff, effective July 1, 2021, providing 8 weeks of paid family leave (at 70% of wages), which will be coordinated with existing leave policies.

^g^ This is correct pending the 2021 update to incorporate a new state paid family and medical leave policy.

^h^ Can use 8 weeks of state paid family leave (maximum $1300 weekly benefit).

^i^ Can use 10 to 12 weeks of state paid family leave (maximum $840 weekly benefit).

^j^ Requires enrollment in supplemental STD plan within 30 days of hire or pregnancy. May be subject to preexisting condition status if enrolled during future open enrollment period. Eligible for benefits after 12 months of active employment.

^k^ Can take 10 to 12 weeks state paid family medical leave (maximum $1000 weekly benefit).

^l^ Information obtained from website. Human resources or benefits department would not confirm.

The mean duration of paid leave for birthing mothers (childbirth plus parental leave) was 7.8 weeks (range, 0-14 weeks), and that for parental leave was 3.6 weeks (range, 0-12 weeks), using benefits from primary caregiver and academic faculty status where institutional benefits differ (Figure). Conversely, using benefits from secondary caregiver or nonfaculty status where benefits differ, the mean paid leave for birthing mothers was 6.8 weeks (range, 0-12 weeks), and that for parental leave was 2.3 weeks (range, 0-8 weeks). Although most institutions (23 institutions [85.2%]) offered short-term disability for childbirth, pay varied markedly (range, 0% to 100%). Four institutions (14.8%) did not provide paid leave for childbirth, and more than one-half (15 institutions [55.6%]) did not provide paid parental leave beyond childbirth (according to secondary caregiver or nonfaculty status where benefits differ).

**Figure.**
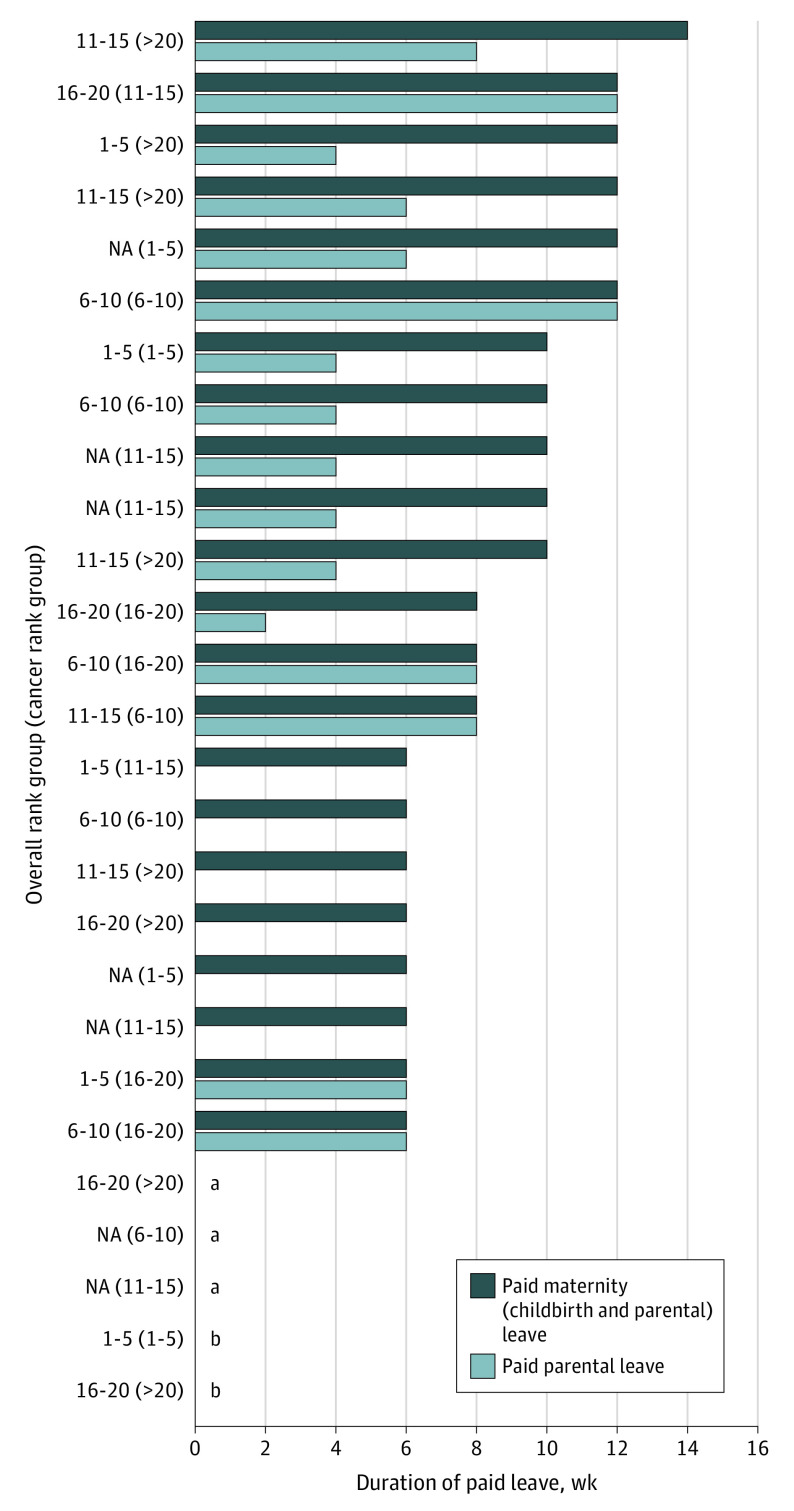
Paid Parental Leave at* U.S. News & World Report* 2020-2021 Best Hospitals and Best Hospitals for Cancer Graph shows total maternity (childbirth plus parental) leave duration based on uncomplicated vaginal delivery. Estimates shown represent maximal benefits estimates where benefits differ. Hospital rankings are designated by groups of 5 or if more than 20. For the full list of included institutions, see eMethods in the Supplement. ^a^Denotes no paid maternity or parental leave. ^b^The parental leave policy was confirmed to be 0 but the pay level during short-term disability was not disclosed.

## Discussion

Despite longer paid leave being associated with improved health of mothers and infants, career satisfaction among female physicians, favorable societal economic impact, and sustained lactation and breastfeeding,^1,5^ the mean duration of paid maternity and parental leave at leading US hospitals and cancer centers is only 7.8 weeks and 3.6 weeks, respectively, at maximal benefits estimates. In contrast, the mean duration of paid maternity leave among other Organisation for Economic Co-operation and Development countries is 18.6 weeks (13.6 weeks at 100% pay).^6^

Several limitations should be considered. Because only *U.S. News & World Report* top-ranked institutions were analyzed, the results may not be generalizable to other institutions. We did not evaluate unpaid leave duration or department-specific policy variations, nor did we assess the association of family leave with outcomes. These results highlight a striking variation in paid parental policies among leading US hospitals and cancer centers, generally well below American Academy of Pediatrics recommendations and Organisation for Economic Co-operation and Development benchmarks, suggesting that evidence-based updates that inclusively support the health and well-being of families and gender equity in the workplace should be considered.

## Notes

Supplement. eMethods.
