# Supplementary material for: Paid Parental Leave Policies Among U.S. News & World Report 2020-2021 Best Hospitals and Best Hospitals for Cancer
Source: JAMA Netw Open. 2021 May 11;4(5):e218518. doi: 10.1001/jamanetworkopen.2021.8518 (PMC8114142; doi:10.1001/jamanetworkopen.2021.8518)
Supplement: Supplement. — eMethods. [file jamanetwopen-e218518-s001.pdf]

Supplemental Online Content

Lu DJ, King B, Sandler HM, Tarbell NJ, Kamrava M, Atkins KM. Paid parental leave policies among *U.S. News & World Report* 2020-2021 best hospitals and best hospitals for cancer. *JAMA Netw Open*. 2021;4(5):e218518. doi:10.1001/jamanetworkopen.2021.8518

eMethods.

This supplemental material has been provided by the authors to give readers additional information about their work.

© 2021 Lu DJ et al. *JAMA Network Open.*

eMETHODS

Family and Medical Leave Act (FMLA) was not included as it represents job-protected unpaid leave.

The following institutions were included on the basis of the 2020-2021 *U.S. News & World Report* Top 20 Best Hospitals and Best Hospitals for Cancer, in alphabetical order.

A. Barnes-Jewish Hospital / Siteman Cancer Center
B. Brigham and Women's Hospital / Dana-Farber/Brigham and Women's Cancer Center
C. Cedars-Sinai Medical Center
D. City of Hope Comprehensive Cancer Center
E. Cleveland Clinic
F. H. Lee Moffitt Cancer Center and Research Institute
G. Hospitals of the University of Pennsylvania-Penn Presbyterian
H. Houston Methodist Hospital
I. Johns Hopkins Hospital
J. Keck Hospital of USC / USC Norris Cancer Center
K. Massachusetts General Hospital
L. Mayo Clinic- Rochester
M. Mayo Clinic-Phoenix
N. Memorial Sloan Kettering Cancer Center
O. Mount Sinai Hospital
P. New York-Presbyterian Hospital-Columbia and Cornell
Q. Northwestern Memorial Hospital
R. NYU Langone Hospitals
S. Roswell Park Comprehensive Cancer Institute
T. Rush University Medical Center
U. Seattle Cancer Care Alliance/University of Washington Medical Center
V. Stanford Health Care-Stanford Hospital
W. UCLA Medical Center
X. UCSF Medical Center
Y. University of Michigan Hospitals-Michigan Medicine
Z. University of Texas MD Anderson Cancer Center
AA. UPMC Presbyterian Shadyside

© 2021 Lu DJ et al. *JAMA Network Open.*
